# Acute or chronic pulmonary emphysema? Or both?—A contribution to the diagnosis of death due to violent asphyxiation in cases with pre-existing chronic emphysema

**DOI:** 10.1007/s00414-021-02619-7

**Published:** 2021-06-28

**Authors:** Giuseppe Gava, Simon B. Eickhoff, Timm J. Filler, Felix Mayer, Nina S. Mahlke, Stefanie Ritz-Timme

**Affiliations:** 1 Institute of Legal Medicine, Heinrich Heine University Düsseldorf, Düsseldorf, Germany; 2 Institute of Systems Neuroscience, Medical Faculty, Heinrich Heine University Düsseldorf, Düsseldorf, Germany; 3 Institute of Neuroscience and Medicine, Brain & Behaviour (INM-7), Research Centre Jülich, Jülich, Germany; 4 Institute for Anatomy I, Heinrich Heine University Düsseldorf, Düsseldorf, Germany

**Keywords:** Acute versus chronic pulmonary emphysema, Violent asphyxiation, Aquaporin 5, Surfactant protein A1, Transmission electron microscopy

## Abstract

The diagnosis of death due to violent asphyxiation may be challenging if external injuries are missing, and a typical acute emphysema (AE) “disappears” in pre-existing chronic emphysema (CE). Eighty-four autopsy cases were systematically investigated to identify a (histo-) morphological or immunohistochemical marker combination that enables the diagnosis of violent asphyxiation in cases with a pre-existing CE (“AE in CE”). The cases comprised four diagnostic groups, namely “AE”, “CE”, “acute and chronic emphysema (AE + CE)”, and “no emphysema (NE)”. Samples from all pulmonary lobes were investigated by conventional histological methods as well as with the immunohistochemical markers Aquaporin 5 (AQP-5) and Surfactant protein A1 (SP-A). Particular attention was paid to alveolar septum ends (“dead-ends”) suspected as rupture spots, which were additionally analyzed by transmission electron microscopy. The findings in the four diagnostic groups were compared using multivariate analysis and 1-way ANOVA analysis. All morphological findings were found in all four groups. Based on histological and macroscopic findings, a multivariate analysis was able to predict the correct diagnosis “AE + CE” with a probability of 50%, and the diagnoses “AE” and “CE” with a probability of 86% each. Three types of “dead-ends” could be differentiated. One type (“fringed ends”) was observed significantly more frequently in AE. The immunohistochemical markers AQP-5 and SP-A did not show significant differences among the examined groups. Though a reliable identification of AE in CE could not be achieved using the examined parameters, our findings suggest that considering many different findings from the macroscopical, histomorphological, and molecular level by multivariate analysis is an approach that should be followed.

## Introduction

Death by violent asphyxia can be caused by various mechanisms (e.g., strangulation, covering of the external airways), which may cause typical findings. Such findings may include external and internal signs of violence, the so-called congestion syndrome (with petechial hemorrhages) as well as acute emphysema (AE); in addition, there may be further external injuries, such as holding, defense, and/or counter-pressure injuries [1–7].

However, the pattern of findings may also be very discrete. This might be especially the case if the victim was unable to defend him- or herself (e.g., in cases of physical superiority of the offender, intoxication of the victim, or physically frail victims like children or elderly [7–11]). Other reasons for missing skin or tissue findings, like hematoma or hemorrhages, may be very smooth, soft strangulation tools, or wearing gloves. Other problematic examples are cases of burking (a combination of covered airways and thorax compression [5, 12, 13]) or deaths due to physical restraint [14]. In such cases, the suspicion of violent death can only be substantiated by internal postmortem findings and histological examination [15–22]; otherwise, it becomes a diagnosis by exclusion [7, 17, 23]. Generally, it has to be noted that the many common findings are only typical, but not specific for violent asphyxiation [3, 4, 24, 25] and may also occur with other causes of death.

Much research has focused on the pulmonary findings. Brinkmann et al. described a specific combination of emphysema, microembolism syndrome, alveolar septal edema, and hemorrhagic-dysoric syndrome as “pathognomonic of obstructive asphyxia” [17]. However, this conclusion was refuted by other authors, since this constellation of findings was also found in control groups, e.g., in cases of shock or resuscitation [26, 27]. Several studies on human lung tissue described a significant increase in alveolar macrophages and giant cells, especially in protracted asphyxia [10, 28–31]. Betz et al. [32, 33] and Grellner et al. [34], conversely, described in their studies that there is no significant giant cell formation or alveolar macrophage proliferation in deaths by asphyxia, which could also be substantiated in 2019 by Gutjahr et al. [27, 35] (“pre-existence hypothesis”). The agony times in these studies correspond to the “realistic” times in the cases of asphyxiation in everyday forensic medicine.

A very relevant pulmonary finding is the acute pulmonary emphysema or acute alveolar dilation, respectively [3, 5]. In some cases, an interstitial emphysema is described [6, 36]. There are only a few differential diagnoses to be considered (e.g., resuscitation or mechanical ventilation [37], severe asthma [38]), which in most cases can be easily excluded by the patient’s history. In young, primarily healthy people, it can usually be clearly diagnosed by macroscopical and histological examination. However, diagnostic problems regularly arise in patients with pre-existing chronic emphysema (CE) [39, 40]. In these cases, the AE may be “overlayed” and cannot be distinguished anymore, especially in collapsed lungs after regular autopsy [41]. There is currently no diagnostic marker or morphological finding that enables the diagnosis of “AE in CE”. Many studies on the diagnosis of violent asphyxiation did not include or explicitly exclude cases of CE or AE + CE [42–51].

Regarding the significantly different pathomechanisms of the development of AE and CE, the identification of “AE in CE” should be possible. While AE develops within minutes due to sudden mechanical stress [1, 52], chronic emphysema develops over several years due to a variety of causes (e.g., senile emphysema [53–55], chronic obstructive pulmonary disease (COPD) [56], alpha-1-antitrypsin deficiency [57], or other secondary forms [58]). Whereas asphyxiation leads to acute hyperinflation and acute ruptures in the alveolar septums, remodelling processes in the elastin and collagen structure have already occurred in chronic emphysema [59–61].

Against this background, 84 autopsy cases in four diagnostic groups (“AE”, “CE”, “AE + CE”, “NE” (no emphysema)) were systematically investigated to identify a specific histomorphological or macroscopical constellation of findings or an immunohistochemical pattern that enables the diagnosis “AE in CE”. Samples from all pulmonary lobes were investigated by conventional histological methods (hematoxylin–eosin, Elastica-van-Gieson, and iron staining) as well as with immunohistochemical markers Aquaporin 5 (AQP-5) and Surfactant protein A1 (SP-A) that have been described as being significantly differentially expressed in the lungs of asphyxiation victims than in other causes of death [42–51, 62]. Particular attention was paid to alveolar septum ends (“dead-ends”) suspected as rupture spots. These were additionally analyzed by transmission electron microscopy (TEM). The histomorphological and macroscopical findings in the four diagnostic groups were compared and evaluated by a multivariate approach, based on machine learning. Importantly, evaluation of the models was performed by out-of-sample prediction, that is, we assessed, how accurately the model could predict the different diagnoses in new, previously unseen cases, i.e., in subjects that have not been part of the training set. The AQP-5 and SP-A results were assessed separately.

## Material and methods

### Case selection, autopsy specimens, and macroscopical findings

All cases were retrospectively selected by checking the autopsy reports of the Institute of Legal Medicine in Duesseldorf (Germany). A total of 84 cases (51 male and 33 female individuals with ages between 1 day and 90 years) were selected based on previous history, police investigations, and reported macroscopic findings, and divided into four groups according to the diagnosis of pulmonary emphysema. The groups were “acute emphysema = AE” (*n* = 22), “chronic emphysema = CE” (*n* = 43), “acute + chronic emphysema = AE + CE” (*n* = 12), and “no emphysema = NE” (*n* = 7). We categorized our cases primarily according to the form of emphysema and did not further subdivide the groups according to the forms of asphyxia since we wanted to address the situation “suspected asphyxiation/suffocation, discrete external findings, unclear course of events”. Individuals with evidence of violent asphyxiation or resuscitation and a clear emphysema without any indication of pre-existing emphysema were assigned to the “AE” group. Individuals with evidence of pre-existing emphysema (e.g., known COPD) or macroscopically visible pulmonary hyperinflation (e.g., due to senile emphysema, which is often an incidental finding), but without clues for violent asphyxiation or resuscitation, were assigned to the “CE” group. The group “AE + CE” comprised individuals with described pre-existing CE and assumed AE due to the cause of death (asphyxiation) that was based on other findings (e.g., external injuries) and case history. Individuals without pathological lung changes were assigned to the “NE” group. Exclusion criteria were signs of advanced putrefaction or severe lung diseases (other than chronic emphysema) such as pneumonia or tumors. Since acute emphysema can also occur during resuscitation with artificial ventilation [37], we included these cases in the “AE” group. A performed resuscitation was also not an exclusion criterion in the “AE + CE” group; however, this did not occur in our cases. The delay between the time of death and performing autopsy varied between 0 and 17 days. In 21 cases, the exact interval was unknown.

For each case, the macroscopical findings documented in the autopsy protocols were assessed and coded in numbers for statistical analysis, as shown in Table 1.

**Table 1:** Macroscopic findings documented from the autopsy protocols for multivariate analysis

Numbers used for coding the findings for statistical analysis		0	1	2	3	4	5	6	7	In gram [g]
External findings	Resuscitation	Not performed	Performed							
	Putrefaction	Not present	Mild, not affecting the lungs							
Lung findings	Right lung weight									x
	Left lung weight									
	Condition of the left pleura	Intact	Lacerated	Stucked on chest wall	Fused with chest wall					
	Condition of the right pleura									
	Consistency of lung tissue	Normal	Increased pressure resistance	Over-inflated	Massively over-inflated					
	Color of the cut surface	Red-livid	Pale red	Dark red	Multiple bloody areas					
	Fluid content	Normal	Mild discharge of liquid	Strong discharge of liquid						
	Blood congestion	None	Mild	Severe						
	Hemorrhages	Not present	Present							
	Signs of inflammation	None	Slight signs of inflammation	Markedly inflamed/pus	Abscess					
	Rupture strength	Normal	Slightly reduced							
	Focal findings	Not present	Present							
	Pulmonary artery sclerosis	Not present	Present							
	Content of pulmonary arteries	Normal blood	Thrombus							
	Bronchial contents	Normal air	Bloody mucus	Brown mucus	Foamy content	Clear secretion	Turbid/milky mucus	Aspirates	Yellowish, purulent mucus	
Heart findings	Dilatation of the right ventricle	Not dilated	Slightly dilated	Strongly dilated						

### Lung tissue samples

A peripheral sample from each upper and lower lung lobe and a central sample from the right middle lobe was taken. They were stored in 4% phosphate-buffered formaldehyde solution at room temperature. For light microscopic and immunohistochemical staining, they were further embedded in paraffin and sliced into 2 µm thick sections and then stored at room temperature until staining (see below). For electron microscopic preparation, see below.

### Conventional histology

The specimens from all lung lobes were stained according to standard protocols for hematoxylin–eosin (H&E) and Elastica-van-Gieson (EvG) [63]. The right lower lobe was additionally stained in Berlin blue iron staining (Fe) to distinguish hemosiderin-containing macrophages (siderophages), which are an indirect sign of chronic heart failure [64], from regular alveolar macrophages. Histological findings that were described as typical for violent asphyxia [20, 21, 26] were recorded in a standardized form. In addition, we evaluated and classified the blind-ending alveolar septal ends, which we have called “dead-ends”. When examining the lung specimens, we deliberately decided against screening them according to strictly defined, side-by-side visual fields in the specimen, since it can occur that a large part of the observed area consists only of an atelectasis, an emphysema bubble (bulla), a large (central) vessel, or some other large-scale change. To get an initial overview of the specimen, we first viewed it at a low magnification (× 40). Then, at a higher magnification (× 200–400), we examined the “representative” areas of the specimen, i.e., those without the above-mentioned changes. We documented the findings as shown in Table 2. The specimens were assessed separately by two examiners.

**Table 2:** Histological findings. In hematoxylin–eosin stained sections, all shown findings except the siderophages were assessed semi-quantitatively in the entire specimen. Particularities were documented qualitatively. In Elastica-van-Gieson stained sections only the “dead-ends” and particularities were documented, and in the Berlin blue stained sections only the siderophages and particularities

		semi-quantitative				qualitatively assessed
		−	+	+ +	+ + +	
Atelectasis		none	mild	severe		
Alveolar dilatation		none	mild	severe		
Edema	interstitial	none	mild	severe		
	alveolar	none	mild	severe		
	alveolar	none	mild	severe		
Hemorrhages	interstitial	none	mild	severe		
	periarterial	none	mild	severe		
	peribronchial	none	mild	severe		
Hyperemia		none	mild	severe		
Alveolar macrophages		none	few	many	many in clusters	
Siderophages		none	few	many	many in clusters	
Dead-ends	smooth	none	few	many		
	fringed	none	few	many		
	drumstick-like	none	few	many		
Pulmonary artery sclerosis		not present	present			
Pathological vessel content		not present	present			
Particularities						

### Immunohistochemistry

We used the following polyclonal primary antibodies for immunohistochemistry (IHC):

- Aquaporin 5 (AQP-5), rabbit (ABIN731260, obtained via www.antibodies-online.com)
- Surfactant Protein A1 (SP-A), rabbit (ABIN3187728, obtained via www.antibodies-online.com)

For AQP-5 staining, we selected specimens from the right upper or lower lobe that showed a preserved bronchial epithelium in H&E staining for internal positive control, resulting in a total of 43 cases (18 “AE”, 11 “CE”, 8 “AE + CE”, and 6 “NE”). For SP-A, we used the right upper and lower lobe from 78 cases (18 “AE”, 42 “CE”, 11 “AE + CE”, and 7 “NE”). Cases in which the AE was probably only caused by resuscitation procedures were excluded because the changes described in the literature were only described for death by asphyxia and not for acute emphysema alone.

The sections were dewaxed with xylene and a descending alcohol series before rehydrating them in distilled water. Afterwards, we demasked the epitopes by incubating them in citrate buffer (pH 6.0) for 45 min in a steam cooker. For staining, they were first incubated in peroxidase block solution (Cell Marque™) for 10 min. Then, 300-fold diluted primary antibody solution was added (200µL on every section), and the samples were incubated at 4 °C in the humidity chamber overnight. Then, the secondary antibody (Histofine® Simple Stain MAX PO) was added, followed by incubation of 30 min. The AQP-5 sections were incubated in DAB (3,3′-diaminobenzidine) chromogen for 10 min and the SP-A sections in AEC (3-amino-9-ethylcarbazole) chromogen for 15 min, respectively. After every incubation, the samples were washed three times in TBST (Tris-buffered saline with Tween®) buffer. Finally, they were counterstained with hematoxylin for about 20 s before covering them with Aquatex®. As positive controls, we used human kidney specimens from one of our autopsy cases for both primary antibodies. Negative controls for each slide were processed according to the described staining protocol without adding the primary antibodies.

The AQP-5 immunoreactivity in the bronchial epithelium was assessed as follows: negative ( −), weakly positive ( +), and strongly positive (+ +). The findings in pneumocytes type I cells were negative ( −), some single cells positive ( +), and positive with a linear pattern (+ +).

For the evaluation of SP-A immunostaining, we used the same classification according to Zhu et al. [44, 65]. Pneumocytes type II cells and alveolar surface (membranous or linear pattern): negative ( −), weakly positive ( +), diffusely, and clearly positive (+ +), and strongly positive (+ + +). Intra-alveolar SP-A aggregates (granular pattern): negative ( −), a few aggregates in some alveoli ( +), some bigger aggregates in some alveoli (+ +), and many massive aggregates in almost all alveoli (+ + +). We always documented the findings for those areas that showed the strongest pattern. The specimens were also assessed separately by two examiners.

### Transmission electron microscopy

The formalin-fixed tissue from six “AE” and five “CE” cases was refixed overnight in 4% glutaraldehyde (GA) in phosphate-buffered saline (PBS) buffer at 4 °C. One lung tissue sample taken prospectively during another autopsy was directly fixed in conventional EM-fixans (2.5% GA, 4% paraformaldehyde in 0.1 M cacodylate buffer, pH 7.4) to compare the image quality between the two methods. 3 × 3 mm cube-shaped specimens were cut out manually for further processing. They were incubated in a 1% osmium tetroxide solution in PBS or 0.1 M cacodylate buffer respectively for 2 h and rinsed in aqua dest before dehydrating them in acetone (30%, 50%, 70%, 90%, and 100%). While dehydration in 70% acetone, block contrast was applied (1% phosphotungstic acid/0.5% uranyl acetate in 70% acetone). Further SPURR embedding medium (Serva, Heidelberg, Germany) was used to embed samples which were then polymerized overnight at 70 °C. We produced 1 µm semi-thin sections and stained them in toluidine blue to search the alveolar septal segments of our interest for further ultrastructural investigation. After this, the samples were cut into 70 nm thin slices by using an Ultracut EM UC7 (Leica Microsystems GmbH, Wetzlar, Germany) and stained with lead-citrate (according to Reynolds [66]) for 8 min and 1.5% uranyl acetate for 25 min. Images were captured using an H-7100 TEM (Hitachi, Tokyo, Japan) at 100 kV and a Morada SIS Camera system; they were subsequently processed by the Olympus ITEM 5.0 Software.

### Statistical analysis and establishment of a multivariate analysis

The macroscopic autopsy findings and the histological findings recorded in the standardized form were analyzed using multivariate pattern recognition. This approach builds a model predicting individual diagnosis on the training sample that is then applied to the previously unseen test data. In more detail, we considered the information from macro- and microscopy as the features on which a classification algorithm is trained to predict the diagnosis, i.e., the results of the autopsy, serving as the target variable. As learning the relationships between features and targets obviously requires known diagnoses, the ability of the thus trained algorithms to correctly diagnose new subjects needs to be tested on new cases for which the model is provided with only the features and the ensuing diagnosis is then evaluated against the known (to us but not the algorithm) true diagnosis. Here we performed such evaluation by a standard leave-one-out approach, i.e., each individual case was subsequently removed from the data before the model was trained on the remaining cases. The trained model is then applied to the features of the held-out subject and the decision recorded. For the actual prediction model, we employed boosted decision trees, as a widely used ensemble model incrementally aggregating binary decision trees by focussing each new iteration on those instances (within the training sample) that were previously miss-classified. Here, we used the implementation within Matlab R2020a with the following settings: total boost algorithm, maximum of 6 splits per tree, margin precision 0.005.

The mean occurrence of the dead-ends in EvG sections and alveolar macrophages and the mean expression of the SP-A patterns were separately evaluated in a 1-way ANOVA analysis and presented as boxplots. *p*-values < 0.05, corrected for multiple comparisons using the false discovery rate (FDR), were considered significant.

## Results

### Multivariate analysis from macroscopic and conventional histological pulmonary findings

All conventional histological findings (described as typical pulmonary findings for violent asphyxia in the literature, see Table 2) could be observed in our cases, although to varying extents.

Our multivariate model derived from all conventional histological and macroscopic findings was able to correctly predict the correct clinical diagnoses in new cases, i.e., those that have not been seen during training with an accuracy of 0.79 (balanced accuracy 0.7, F1-Score 0.82).

**Fig. 1:**
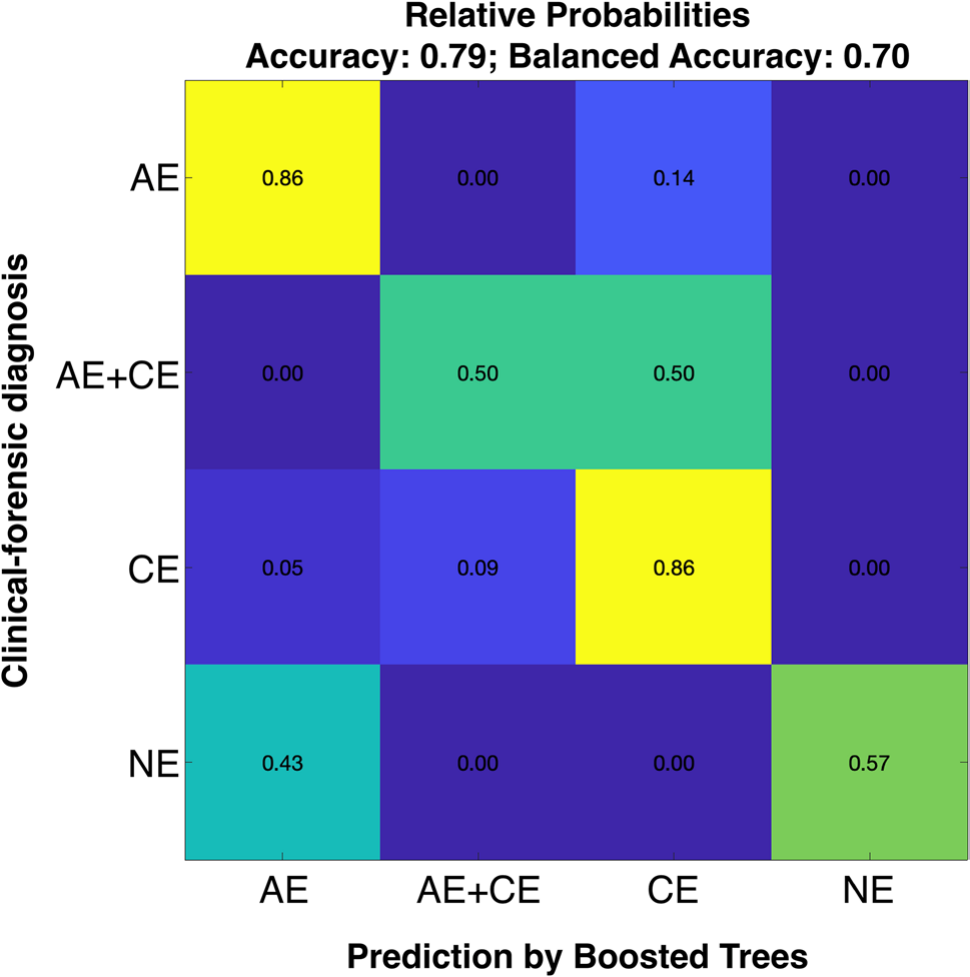
Results of the multivariate analysis including all light microscopic and macroscopic findings. Prediction probabilities (numbers in the boxes) denoting how likely a given clinical-forensic diagnosis was assigned correctly to a particular label in the algorithm evaluation (Accuracy = number of correctly classified cases / all cases, Balanced Accuracy: mean accuracy for each individual diagnostic group, AE = acute emphysema, CE = chronic emphysema, AE + CE = acute and chronic emphysema, NE = no emphysema)

In detail, the different diagnoses could be predicted with the following probabilities (Fig. 1):

- 86% probability for a correct classification of “AE” cases as “AE” cases
- 86% probability for a correct classification of “CE” cases as “CE” cases
- 57% probability for a correct classification of “NE” cases as “NE” cases
- 50% probability for a correct classification of “AE + CE” cases as “AE + CE” cases
- 50% probability for a false classification of “AE + CE” cases as “CE” cases
- 43% probability for a false classification of “NE” cases as “AE” cases
- 14% probability for a false classification of “AE” cases as “CE” cases
- 9% probability for a false classification of “CE” cases as “AE + CE” cases
- 5% probability for a false classification of “CE” cases as “AE” cases
- Each 0% probability for a false classification of “AE” cases as “AE + CE” cases, “AE” cases as “NE” cases, “AE + CE” cases as “AE” cases, “AE + CE” cases as “NE” cases, “CE” cases as “NE” cases, “NE” cases as “AE + CE” cases, and “NE” cases as “CE” cases, respectively.

### Special histological findings

**Fig. 2:**
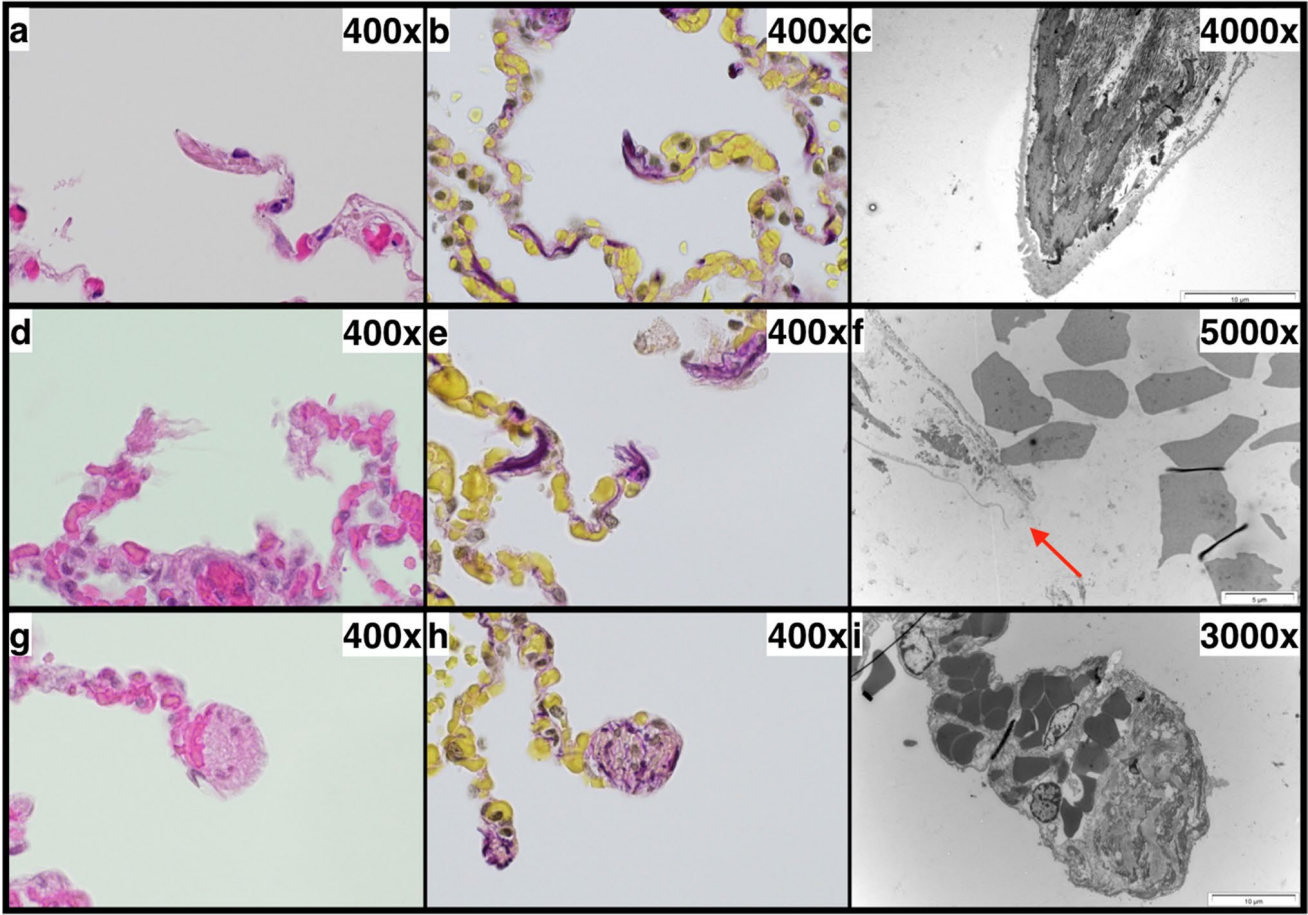
Examples for the observed types of dead-ends: “smooth” dead-ends (**a**–**c**), “fringed” dead-ends (**d**–**f**), and “drumstick-like” dead-ends (**g**–**i**) (**a**, **d**, **g** = hematoxylin–eosin stain; **b**, **e**, **h** = Elastica-van-Gieson stain; **c**, **f**, **i** = transmission electron microscopy; * = erythrocytes; arrow = membrane defect. **b**, **d**, **e**, **g**–**i** = cases with acute emphysema; **a**, **c**, **f** = cases with chronic emphysema)

Three types of dead-ends (Fig. 2) could be identified:

- “Drumstick-like” dead-ends with a pronounced rounded thickening at the tip
- “Fringed” dead-ends with an irregularly shaped tip, appearing destroyed
- “Smooth” dead-ends with a smooth and continuously membrane-covered tip

**Fig. 3:**
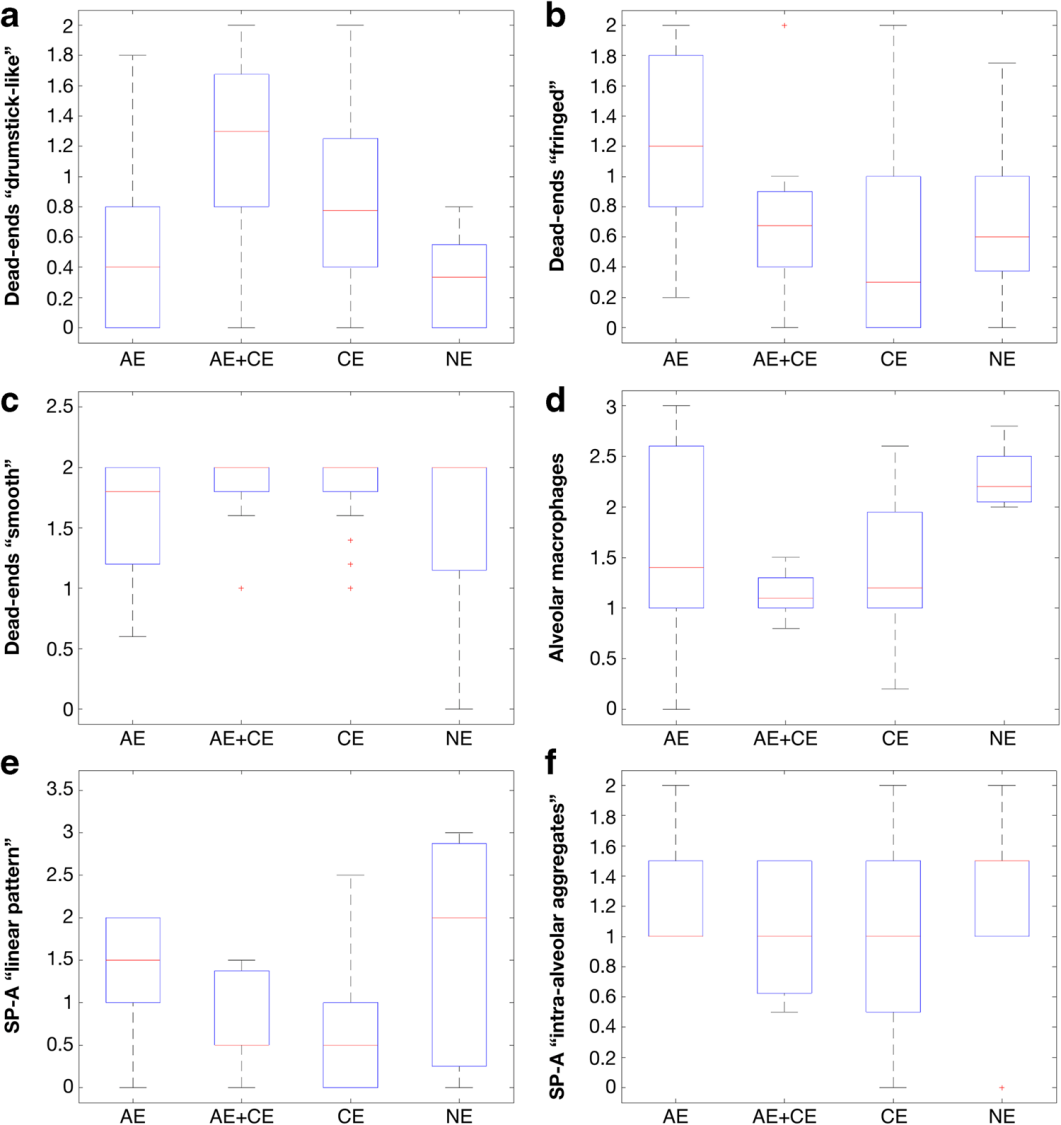
1-way ANOVA analysis. The data sum up the findings from all pulmonary lobes. Boxplots show median and inter-quartile ranges. *p*-values < 0.05, corrected for multiple comparisons using the false discovery rate (FDR), were considered significant. **a** Mean occurrence of “drumstick-like” dead-ends (from “0” = “none” to “2” = “many”). Significant difference between the “AE + CE” and the “NE” group (*p* = 0.006). **b** Mean occurrence of “fringed” dead-ends (from “0” = “none” to “2” = “many”). Significant difference between the “AE” and the “CE” group (*p* < 0.001). **c** Mean occurrence of “smooth” dead-ends (from “0” = “none” to “2” = “many”). **d** Mean occurrence of alveolar macrophages (from “0” = “none” to “3” = “many in clusters”). Significant differences between the “AE + CE” and the “NE” group (*p* < 0.001) and between “CE” and “NE” (*p* < 0.001). **e** Mean expression of the linear SP-A pattern (from “0” = “negative” to “3” = “strongly positive”). **f** Mean occurrence of intra-alveolar SP-A aggregates (from “0” = “negative” to “3” = “many massive aggregates in almost all alveoli”) (AE = acute emphysema, CE = chronic emphysema, AE + CE = acute + chronic emphysema, NE = no emphysema, SP-A = surfactant protein A)

“Drumstick-like” dead-ends were significantly more present in the “AE + CE” group than in the “NE” group (*p* = 0.006, Fig. 3a). This type of dead-end was also observed frequently in the “CE” group (Fig. 3a); however, the difference between the “CE” group and the other groups was not significant.

“Fringed” dead-ends were significantly more frequently seen in the “AE” group than in the “CE” group (*p* < 0.001, Fig. 3b). They were also observed more frequently in the “AC + CE” group than in the “CE” cases; however, this difference was not significant.

There were no significant differences in the “smooth” dead-ends among the groups (Fig. 3c).

Alveolar macrophages were significantly less frequently observed in the “CE” group and in the “AE + CE” group than in the “NE” group (*p* < 0.001, Fig. 3d).

### TEM

The formalin-fixed material exhibited a sufficiently good image quality compared to the fresh lung tissue fixed directly during autopsy in conventional fixatives for electron microscopy. Thus, it was possible to examine tissue that was preserved up to 2 years ago.

**Fig. 4:**
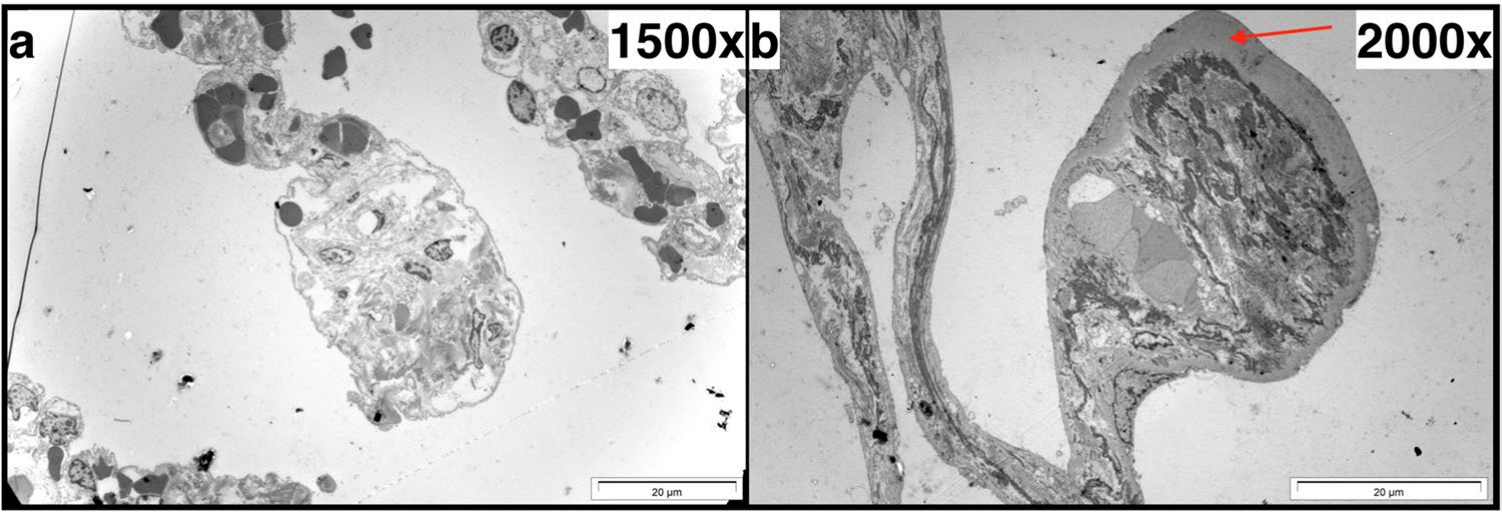
Two examples for different “drumstick-like” dead-ends in transmission electron microscopy. **a** Acute emphysema (girl, age of 8 years). **b** Chronic emphysema (woman, age of 46 years). Thick, homogeneous submembrane layer at the tip of the dead-end (arrow)

All three types of dead-ends could be detected (Fig. 2), in “AE” as well as in “CE” cases. The morphological variability of all types of dead-ends was very high. For example, at the tip of some “drumstick-like” dead-ends, a thick, homogeneous submembranous layer could be observed (Fig. 4), especially in cases of CE. In the “AE” cases, we did not found this layer in any of three cases with detectable “drumstick-like” dead-ends, whereas in the “CE” cases, we found it in two out of four cases analyzed by TEM.

### AQP-5 IHC

**Fig. 5:**
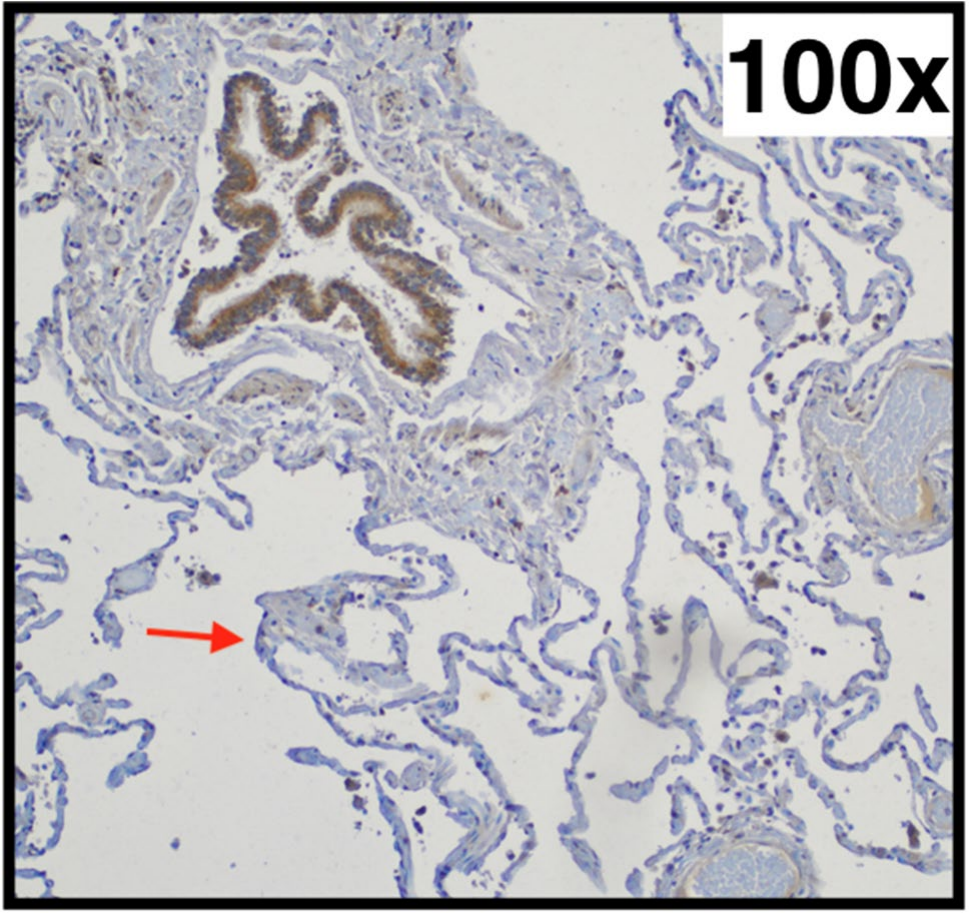
Typical pulmonal findings in AQP-5 (aquaporin-5) immunohistochemistry (right lower lobe, 73-year-old woman with chronic emphysema): Strongly positive bronchial epithelium (upper left corner) next to negative pneumocytes type I (exemplary marked with an arrow)

In all investigated cases, the bronchial epithelium exhibited a strong expression of AQP-5 (internal positive control). However, the pneumocytes type I did not show a clear stainability in any case in all investigated groups (Fig. 5). The external positive and negative controls showed clear or missing stainability with AQP-5, respectively.

### SP-A IHC

**Fig. 6:**
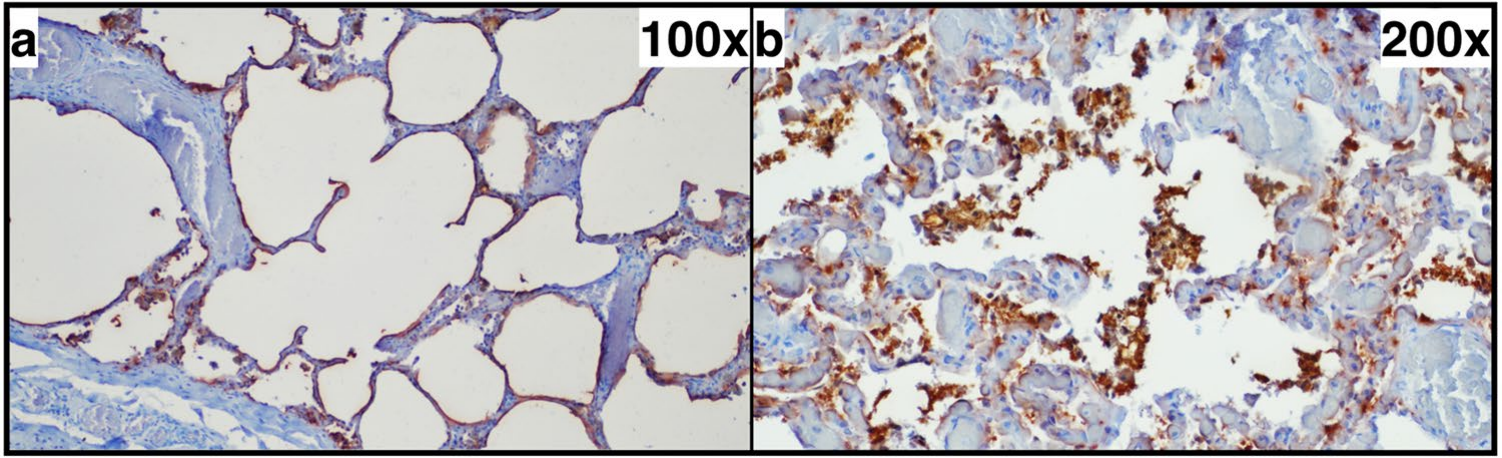
Typical pulmonal findings in SP-A (surfactant protein A) immunohistochemistry. **a** Strong linear pattern (+ + +) in a lung without emphysema of a 30-year-old man. **b** Strong intra-alveolar SP-A aggregates (+ + +) in a lung without emphysema of a 27-year-old man

**Fig. 7:**
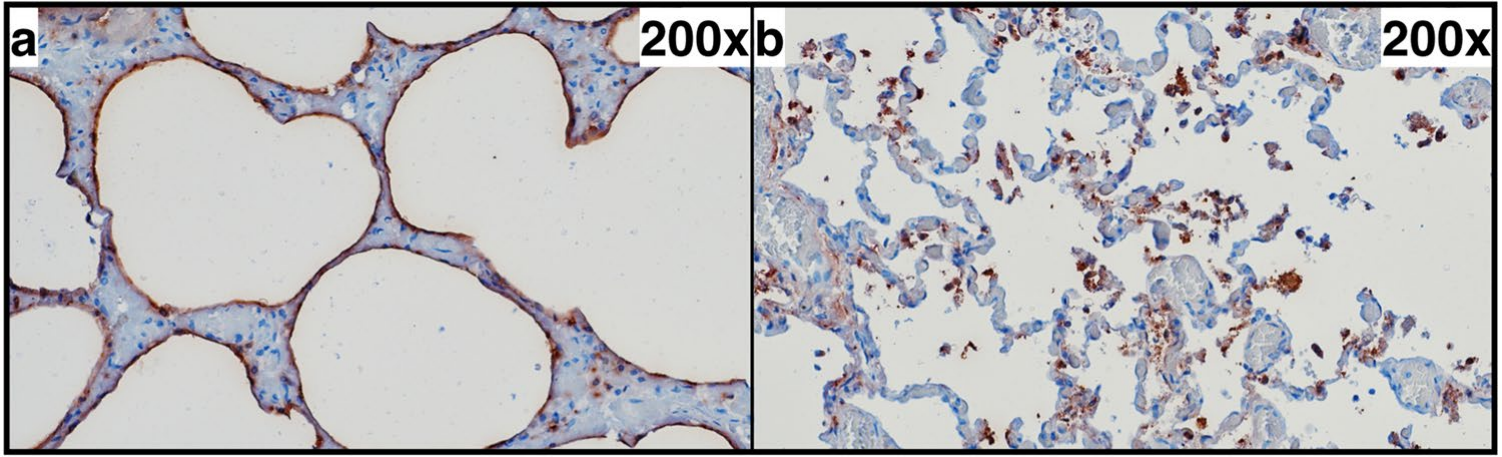
SP-A immunohistochemistry. Both a linear pattern (**a**) and intra-alveolar aggregates (**b**) in the identical case but in different regions. Left upper lobe of a non-emphysematic lung from a 30-year-old man

In principle, the SP-A patterns described in the literature could be reproduced in our samples (Figs. 6, 7). However, the marker did not show significantly different types of expression (linear pattern, intra-alveolar aggregates) between the groups (Fig. 3e, f). Even a separate examination of the right upper and lower lobe did not reveal any significant differences. The external positive and negative controls showed clear or missing stainability with SP-A, respectively; the internal control (smooth vascular muscle) was clearly positive in each case.

## Discussion

### No specific pulmonary findings that would allow the diagnosis of “violent asphyxia” in cases with pre-existing CE

Despite the extensive examination of numerous findings that are supposed to be typical for violent asphyxia, we could not identify any specific finding that would allow a reliable diagnosis in cases with acute and pre-existing chronic emphysema (“AE + CE”).

The significantly more frequent occurrence of alveolar macrophages in the “NE” group compared to the “CE” and “AE + CE” groups contradicts our expectations. When interpreting the data, the numerous factors that influence the number of macrophages, like COPD [67, 68], must be considered. The agony time achieved in reality does not seem to provide a significantly increased number in cases of asphyxia, as already described by other authors like Gutjahr et al. [27].

With the exception of alveolar macrophages and dead-ends (see below), we consciously decided against a statistical evaluation of each light microscopic parameter in the 1-way ANOVA analysis separately. The reasons for this are firstly, that our current focus was the possible diagnosis of acute emphysema in cases with pre-existing chronic emphysema (which was an exclusion criteria in previous studies), so it is a pilot study with a low case number (statistical inconclusive), and secondly, that it is already known from the literature that the findings are not specific but only typical for violent asphyxiation.

Interstitial emphysema is described [6, 36] as a possible finding, in the older literature as a finding arising with prolonged asphyxia [69]. In our cases, we could not detect this, which is not in conflict with the literature, as it is not described as a constantly occurring parameter.

### The approach of a multivariate analysis of findings was not successful for the “AE + CE” group in this study but may nevertheless be interesting for future research

The major advantage of the leave-one-out cross validation we used is that model performance can be evaluated with respect to their out-of-sample performance, i.e., we could estimate how well the model is able to correctly classify new cases.

Eighty-six percent of all “AE” and “CE” cases were correctly classified as “AE” and “CE” cases, respectively. Accordingly, the probability of incorrect classification of “AE” and “CE” cases was low (0–14%). The multivariate model correctly classified only 50% of the “AE + CE” cases. The incorrect classified cases “AE + CE” cases were classified as “CE” cases. The unsatisfactory performance of the model in the “AC + CE” group may be caused by the low number of cases in the groups and the variability within a group due to the assignment to a group that was only done based on the type of emphysema, not based on the cause of death.

In any case, our findings suggest that multivariate approaches should be investigated further, albeit with a significantly larger number of cases, precisely defined groups, and taking into account other (immunohistochemical and molecular) parameters with diagnostic relevance. Interesting parameters could be further immunohistochemical markers as the hypoxia-inducible factor 1-alpha (HIF1-α [50]) or altered expression patterns of microRNAs from specific proteins in other organs like the myocardium or brain [70, 71]. Furthermore, markers secreted by alveolar macrophages in a hypoxic environment might be of interest, such as MCP-1 [72–74].

### “Dead-ends” as diagnostic tools?

The “smooth” dead-ends are most likely correlates of the alveolar septums’ physiological shape since they were found in every specimen.

The “drumstick-like” dead-ends are to be interpreted as typical findings in chronic pulmonary emphysema. Their shape has already been described in the literature, for example, as “tennis racquet” or “clubbed”-appearing ends [75] or as “stump-like” alveolar septum ends [76]. Rapello et al. described “formations of drumsticks” in their study about pulmonary emphysema development in rat lungs after 90 days of exposure to methylphenidate [77]. However, it should be noted that these were also found in three childish and youthful lungs (7-, 8-, and 15-year-old individuals) of our cases, respectively, who had no evidence for chronic emphysema, so we do not consider them to be specific. We suggest that they may be the morphological correlate to a thickened ring structure (“basal ring” [78]) at the entrance of the alveoli. The occurrence of the homogeneous subepithelial layer at the tip of the “drumstick-like” dead-ends in adult “CE” cases (Fig. 4) may be a sign of a chronic remodelling process. Clarification of its significance requires further investigations.

The “fringed” dead-ends may be a correlate to alveolar wall ruptures and, therefore, a typical finding in acute emphysema. They are not specific since they were detected also in other forms of emphysema. It seems plausible that they may occur in diverse situations of tissue stress, e.g., during coughing attacks [79, 80]. An advanced morphological categorization or a quantitative evaluation may increase the diagnostic value of “fringed” dead-ends.

### The immunohistochemical markers AQP-5 and SP-A did not reveal results of diagnostic value

AQP-5, a transmembrane protein responsible for water transport and expressed mainly on pneumocytes type I [81–86], has been shown a reduced linear expression pattern in forms of asphyxiation in which the airways were obstructed [49] and in mice lungs after freshwater drowning [87]. However, other studies show no differences between fresh and salt water drowning [51] and that there is an increased expression in rat lungs after drowning [88].

We could not reproduce this staining pattern in any case. We have no explanation for the pneumocyte type I lacking stainability with our used marker for AQP-5, since both the internal and the external positive control were clearly stained. One possible explanation could be that our primary antibodies bind to different epitopes than those used in other studies, such as those by Wang et al. [49] or Hayashi et al. [87].

SP-A is an essential component of the surfactant; it is expressed by pneumocytes type II and Clara cells (meanwhile known as club cells) and reduces the surface tension of the alveoli [89–91]. It has been shown to be significantly more expressed in human and animal lungs after (mechanical) asphyxia and drowning (especially a distinct “granular pattern” is described) [42–48, 50, 51, 62].

In our hands, the immunohistochemical SP-A pattern showed up as we expected from the previous literature. We noticed that the intensity of the same specimen’s findings could vary widely, which is why we only evaluated the areas with the most pronounced findings. We saw in the same specimen that a clear linear pattern could be seen next to distinct intra-alveolar aggregates (Fig. 7). However, it did not occur that both patterns appear at the same spot within one specimen, so it seems plausible that the intra-alveolar aggregates are a “sheared off” linear pattern. The lack of significant differences between the groups may be explained by the high variability of the individual cases within the groups and the small case number.

For both IHC markers, it must be noted that their expression is also dependent on other circumstances. For AQP-5, it could already be shown in animal experiments that the expression decreases in case of an adenovirus infection [92] or lung fibrosis [93]. Besides, the bronchial epithelium assessment may be complicated because, in some cases, it has detached from the bronchial wall, for example, due to suction effects or autolysis [35]. The SP-A pattern is highly influenced by pulmonary edema [94], which can also emerge after death [95]. Less SP-A is supposed to be expressed in COPD patients [96] and CO intoxication [65]. Increased SP-A stainability has been shown, for example, in perinatal aspiration of amniotic fluid, fire victims, and intoxications, e.g., with methamphetamine, organophosphates, or muscle relaxants [43, 47, 65]. Due to the lack of specificity, positive findings must be critically evaluated.

### Limitations of this study

We categorized our cases primarily according to the form of emphysema and did not further subdivide the groups according to the forms of asphyxia since we wanted to address the situation “Suspected asphyxiation/suffocation, discrete external findings, unclear course of events”; the resulting high variability of cases (Table 3) should increase the informative value of this pilot study. However, this variability made the interpretation of our findings partly tricky. The individual cases assignment to the groups was made after considering all available information and to the best of our knowledge. Therefore, the form of emphysema of an individual was therefore always only the expected form of emphysema because the actual form could not be proven with absolute certainty. Further investigations should differentiate between the various forms of asphyxia, considering the partly different definitions in the literature [97], and use a larger number of cases.

**Table 3:** Causes of death within the four diagnosis groups (*AE* acute emphysema, *CE* chronic emphysema, *AE* + *CE* acute + chronic emphysema, *NE* no emphysema)

Group	Cause of death	*n*
AE	Atypical hanging	9
	Burking	1
	Drowning	4
	Fatal aspiration	1
	Oronasal occlusion	1
	Resuscitation	2
	Status asthmaticus	1
	Strangulation	3
AE + CE	Aspiration	1
	Atypical hanging	3
	Drowning	2
	Oronasal occlusion	1
	Strangulation	5
CE	Bleeding	4
	Cardial death	21
	CO intoxication	1
	Craniocerebral injury	2
	Gunshot	1
	Hypothermia	2
	Intoxication	1
	Polytrauma	5
	Sepsis	5
	Viral infect	1
NE	Decapitation	1
	Heart failure	1
	Intoxication	1
	Polytrauma	2
	Sepsis	2

## Conclusion

In summary, we could not identify any specific morphologic finding or pattern that would allow a reliable diagnosis of acute emphysema or death by violent asphyxia, respectively, if chronic emphysema is pre-existing. However, we identified “fringed dead-ends” as an interesting and typical (but not specific) finding in AE that deserves further investigation. Though the multivariate analysis of findings was not successful for the “AE + CE” group in this study, this approach may be interesting for future research. In light of the complexity of violent asphyxiation’s pathophysiology, it seems unlikely to find specific diagnostic parameters. In the absence of specific findings, diagnoses must be based on at best many typical findings. Multivariate approaches may be an interesting tool to support the reliability of diagnoses based on typical but not specific findings. They should be investigated further, using large numbers of cases, precisely defined groups, and taking into account several morphological and molecular parameters with diagnostic relevance.
